# Application of a catalytic combustion sensor (Pellistor) for the monitoring of the explosiveness of a hydrogen-air mixture in the upper explosive limit range

**DOI:** 10.1155/S1463924603000208

**Published:** 2003

**Authors:** M. Krawczyk, J. Namiesnik

**Affiliations:** Chemical Faculty Gdansk University of Technology ul. G. Narutowicza 11/12 Gdansk 80-952 Poland

## Abstract

A new technique is presented for continuous measurements of hydrogen contamination by air in the upper explosive limit range. It is based on the application of a catalytic combustion sensor placed in a cell through which the tested sample passes. The air content is the function of the quantity of formed heat during catalytic combustion of hydrogen inside the sensor. There is the possibility of using the method in industrial installations by using hydrogen for cooling electric current generators.


Journal of Automated Methods & Management in Chemistry
Vol. 25, No. 5 (September–October 2003) pp. 115–122

Taylor & Francis

Taylor & Francis Group

### Application of a catalytic combustion sensor (Pellistor) for the monitoring of the explosiveness of a hydrogen–air mixture in the upper explosive limit range

**M. Krawczyk, J. Namiesnik**

*Chemical Faculty, Gdansk University of Technology, ul. G. Narutowicza 11/12, 80-952 Gdansk, Poland*

*A new technique is presented for continuous measurements of hydrogen contamination by air in the upper explosive limit range. It is based on the application of a catalytic combustion sensor placed in a cell through which the tested sample passes. The air content is the function of the quantity of formed heat during catalytic combustion of hydrogen inside the sensor. There is the possibility of using the method in industrial installations by using hydrogen for cooling electric current generators.*

### Introduction

Different types of gases (including flammable gases) are used in many technological processes. Of these, natural gas and hydrogen especially find wide applications. It seems that the importance of hydrogen as an energy carrier and technological medium will increase. It is connected with increasing concern for the environment as well as for economic reasons. Hydrogen is used in the production of electric energy and not only as an energy carrier.

The high heat conduction of hydrogen ($\lambda = 0.186\,W/(m\,K)$ at 20°C) has led to its general application in current generator cooling systems of 30 to over 200 MW power [1]. Application of hydrogen as a cooling medium increases the efficiency of a power unit by 1%, while its low viscosity has an advantageous effect on decreasing rotor frictional resistance. In addition, the noise level decreases significantly. The heat conduction properties of non-flammable helium are similar, but a twice greater viscosity and economic considerations lead to the use of hydrogen in practice only.

The application of hydrogen is completely safe when not contaminated by air. Hence, every installation requires devices for continuous determination of its purity. Analytical devices used at present operate by measuring the difference in the heat conduction of a reference gas (pure hydrogen) and the tested hydrogen sample contaminated by air. The sensor is made up of two or four fibres, or termistors, connected to an unbalanced Wheatstone bridge [2] and heated by the current passing through them. The bridge unbalance voltage is a quantity proportional to the analyte concentration.

Such a sensor is called a thermal conductivity detector (TCD) [3] or katharometer and has many advantages: it does not cause destruction of the analysed substance, and it is characterized by simple construction, high sensitivity and response linearity in a wide measurement range. It is well suited for measurement of the concentration of one gas in a two-component mixture, but owing to lack of selectivity, it is difficult to analyse multicomponent mixtures.

In the case of devices cooled by hydrogen, e.g. electric generators, filling of the cooling system has to take place in two stages as the hydrogen–air mixture (in the concentration ranges of hydrogen from 4 to 75% by volume) is explosive. Hence, air is first removed by carbon dioxide ($CO_2$) and the $CO_2$ is then replaced with hydrogen. During emptying of the system, hydrogen is removed with $CO_2$ and the $CO_2$ is then dislodged by air.

The presence of $CO_2$ in the mixture, the heat conduction of which is three times greater than that of air, causes the katharometer to significantly decrease hydrogen purity indications.

A sensor, for which the analyte is not one of the components but the whole mixture—fuel gas (hydrogen) and oxidizer (air) mixture, would not have the principal disadvantage of the katharometer, being the effect of additional components on sensor indications. For practical applications, the sensor has to have the following features:

- Construction simplicity.
- Small dimensions.
- Small power supply current.
- High mechanical resistance.
- Short response time.
- Relatively low price.

Sensors using the combustion reaction heat for determination of given components are such sensors. They are applied in the lower explosive limit (LEL) range. In the case of hydrogen, it is a concentration of up to 4% of hydrogen in air by volume. Attempts have been made here to use the standard catalytic combustion sensor for measurements of air concentration in hydrogen in the upper explosive limit (UEL) range, i.e. from 0 to 25% of air in hydrogen, and to determine the purity of hydrogen.

* To whom correspondence should be addressed.
e-mail: adschem@pg.gda.pl

### Experimental

*Pellistor sensor*

A standard sensor is made of two elements: active and passive [4]. The catalytic combustion reaction takes place on the active element, while the passive element is used as a reference compensating for external temperature, pressure and humidity changes. The sensor is connected to a Wheatstone bridge (figure 1).

The heat of reaction causes a change in the resistance of the active element and formation of a bridge unbalance voltage on the output, the value of which is a function of gas concentration in the combustion mixture.

Former sensors were designed to detect methane in the mining industry, but the sensor functioning principle shows that every combustion gas and oxidizer (usually oxygen in air) mixture can be an analytical sample. An example of an exothermic catalytic combustion reaction is as follows:

$$CH_4+2O_2 \overset{cat}{\tau} CO_2+2H_2O+Q_s \quad (1)$$

$$2H_2+O_2 \overset{cat}{\tau} 2H_2O+Q_s, \quad (2)$$

where $Q_s$ is the (standard) heat of combustion and equal to −891 and $-572\,kJ\,mol^{-1}$, respectively [5].

The temperature of the active surface is of utmost importance and depends on the detected gas and catalyst. For example, for methane combustion in air and the application of palladium as a catalyst, it is 400–500°C [6].

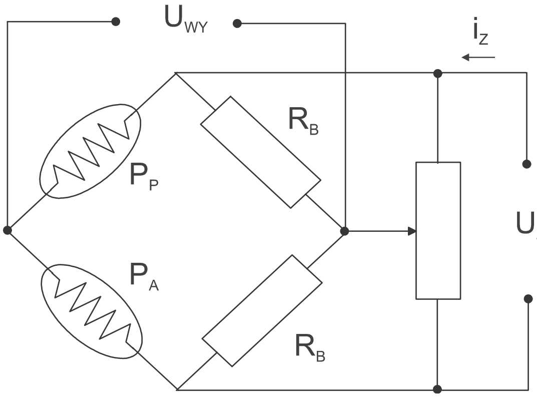

*Figure 1. Catalytic sensor measurement system. $U_Z$, supply voltage of the measurement bridge; $i_Z$, bridge supply current; $U_{WY}$, output voltage; $P_A$, active pellistor; $P_P$, passive pellistor; $R_B$, Wheatstone bridge resistors.*

Two types of universal industrial pellistors were chosen:

- Model 4P-90 from City Technology Ltd [7], belonging to category Ex d IIC, with European [8,9] and Canadian [10] conformity certificates allowing application of this sensor in gas mixture atmospheres creating explosive hazards.
- Model PC-31 from Zakład Elektroniki Górniczej 'ZEG' S.A. [11], also belonging to category Ex d IIC with conformity certificates issued by Główny Instytut Górnictwa — Kopalnia Doświadczalna 'Barbara'.

In real industrial systems, hydrogen purity is evaluated based on measurements with thermal conductivity sensors. Hence, for comparison, a conductivity sensor model PC-32 produced by Zakład Elektroniki Górniczej 'ZEG' S.A. has additionally been used.

In table 1, basic electric and meteorological parameters are presented of sensors used in investigations. The following designations have been assigned to the three types of sensors used: CityTech, ZEG and TCD.

The technical data of sensors refer to determination of methane in the concentration range corresponding to the LEL. Their full description is given in respective technical specifications.

*Experimental stand*

The structure and final form of the measuring set-up was the result of work in the Department of Analytical Chemistry on the application of thermochemical gas sensors [12,13]. The experimental stand was made up of three main elements:

- Generator of standard hydrogen and air gas mixture.
- Analyser measurement cell with pellistor sensor.
- Automatic system for steering and recording of measurement data.

Hydrogen of 5.0 purity degree (99.999% by volume) from Messer [14] was used for investigations. A standard gas mixture stream was produced by the gas mixture generator, the main element of which was a system of mass flow controllers from OMEGA [15]. The system ensured precise measuring of gas components and maintaining a standard gas mixture stream flow at the required level.

Measurements of pellistor signals were performed in a typical system of an unbalanced Wheatstone bridge with a stabilized DC voltage from a laboratory HAMEG HM70042 [16] power supply, with inbuilt $U_Z$ voltage

*Table 1. Basic performance characteristics of industrial pellistors and the thermal conductivity sensor.*

| Sensor | 4P-90 (CityTech) | PC-31 (ZEG) | PC-32 (TCD) |
|---|---|---|---|
| Operating voltage (V) | 3.30 | 3.0 | 2.5 + 3.0 |
| Operating current (mA) | 75 | 60 | 60 |
| Measurement range | 0 + 100% LEL | 0 + 100% LEL | 0 + 100% by vol. |
| Sensitivity (mV/% $CH_4$) | 28 | 25 | 1,5 + 3 |
| Response time $T_{90}$ (s) | < 20 | < 10 | < 10 |

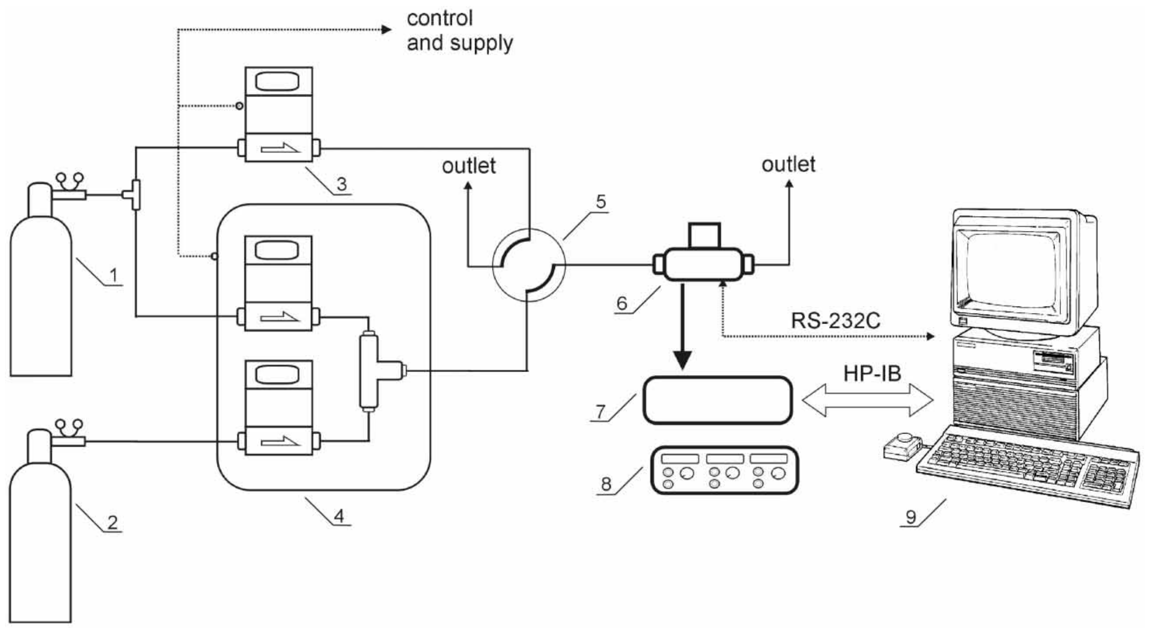

*Figure 2. Measuring set-up. 1, Hydrogen cylinder; 2, air cylinder; 3, mass flow controller; 4, generator of standard gas mixtures; 5, four-way valve; 6, measuring cell with a catalytic sensor; 7, HP 7090A measuring system; 8, laboratory power supply; 9, HP 9000/310 instrument controller.*

and ammeters. The sensor response to the presence of gas mixtures was recorded with a three-channel Hewlett-Packard HP 7090A [17] measurement system connected through an HP-IB (IEEE-48) interface with an HP 9000/310 controller. A schematic diagram of the measuring set-up for determination of gas mixture explosiveness in the UEL range is shown in figure 2.

Steering of the whole set-up was ensured by specialist software written entirely by the authors. In effect, a system of automatic acquisition of data from the measuring set-up has been constructed. The sensor response sampling frequency by the HP 7090A recording system is equal to 500 readings $s^{-1}$ and significantly exceeds measurement requirements. High measuring apparatus parameters could be used to realize real-time software measurement signal filtration in order to reduce the noise level.

### Generator of standard gas mixtures

The gas installation for preparing standard gas mixtures was based on the gas flow mixing technique with the use of mass flow controllers as the main part of the measuring set-up. Application of mass flow controllers imposes requirements about the purity of applied gases. Solid particles especially have to be filtered off beforehand as they can damage the controllers. To ensure stable working conditions and to protect the devices from excess pressure, a two-stage gas reduction system was applied. (Apart from reducing valves on gas cylinders, precise mechanical pressure regulators were built in.) The aim of such a procedure was to obtain a strictly defined constant overpressure of 12 kPa at the inlet to mass flow controllers.

The generator allows blending of two-component mixtures made up of hydrogen and air. In the case of measurements in the UEL range, hydrogen is the zero gas used for determination of the zero point of the sensor and is a matrix (diluent gas) of the prepared standard mixtures. One mass controller is calibrated hydrogen and it has the adjustment range 0–200 $cm^3\,min^{-1}$; the second is calibrated air and allows regulation of the flow in the range 0–10 $cm^3\,min^{-1}$. Calibration of controllers was performed in standard conditions, i.e. at a pressure of 1013.25 hPa and a gas temperature of 20°C.

Controller parameters have been chosen in such a way so as to prepare mixtures of air in hydrogen (from 1 to 6% by volume) and of gas flow in the range 50–200 $cm^3\,min^{-1}$. These quantities result from conditions in which purity sensors are operated in hydrogen cooling power generators. If the air content exceeds 4% by volume, the operators have to make up the cooling system with pure hydrogen delivered from cylinders. It is required for the stream flow of the taken sample to be approximately equal to 150 $cm^3\,min^{-1}$. An excessively small flow of the taken sample would cause a delay (idle time) in the detection of contamination of hydrogen by air while an excessively high flow would lead to unnecessary hydrogen losses in the cooling system.

The concentration of air in hydrogen is calculated from the formula:

$$C = \frac{Q_V^{air}}{Q_V^{H_2} + Q_V^{air}}, \qquad (3)$$

where $C$ is the per cent concentration by volume and $Q_V$ is the air and hydrogen stream flow by volume.

For certainty, it was decided to check the composition of mixtures using an independent measuring technique. A chromatographic technique (GC-TCD) was chosen using Hewlett-Packard HP G1530A equipment. Measurements were performed using a PLOT Q 30 m ×

0.32 mm column (length and diameter) at 50°C. Hydrogen was used as carrier gas ($5\,cm^3\,min^{-1}$).

The constructed standard mixture generator allowed the generation of mixture flows of air content in hydrogen from 1 to 6% by volume for four mixture stream flows: 50, 100, 150 and $200\,cm^3\,min^{-1}$.

### *Investigation of sensor characteristics*

Pellistors applied in investigations are universal sensors allowing detection of flammable gases in air. Manufacturers quote the following sensor parameters in their documentation: power supply voltage, current, sensitivity, response time, etc., only for mixtures in the range up to 5% methane in air (up to 100% LEL). For air mixtures with other gases, these are not optimal parameters but nevertheless are sufficient for correct functioning of the sensor.

In the case of measurements in the UEL range for hydrogen, conditions were totally different due to the physicochemical properties of hydrogen contaminated by air. This results from a higher (by over seven times) heat conduction coefficient by hydrogen in comparison with air, a lower temperature required for catalytic hydrogen combustion and also the non-dimensional excess air factor, $\lambda$, determining the mixture combustion conditions. It is defined as a ratio of the air quantity delivered in a mixture, $L_d$, to the theoretical quantity of air, $L_t$, required for complete combustion of fuel (stoichiometric mixture). This coefficient is given by the following formula:

$$\lambda = \frac{L_d}{L_t}. \tag{4}$$

Optimal combustion conditions for a given gas mixture composition require parameter $\lambda$ to be in the range 1.05–1.20, while the determination of hydrogen contaminated by air, for example for 5% of UEL, causes the air in the hydrogen coefficient to be $\lambda = 0.005$. This value illustrates differences in the operating conditions of a catalytic combustion sensor in the UEL and LEL ranges.

Additionally, intense cooling of the pellistor platinum fibre by hydrogen occurs. In effect, in spite of a lower than standard supply voltage, a higher current flows through the sensor exposed in a hydrogen atmosphere than through a sensor placed in air.

Hence, determination of the effect of power supply voltage and the gas stream flow on sensor dynamic parameters becomes a basic problem. Differences in construction led to the adoption of the following supply voltage for the ZEG pellistor in investigations: $U_Z = 1.5$, 1.7, 1.9 and 2.1 V, while for the CityTech sensor, the following $U_Z$ voltages were used: 2.8, 3.0, 3.2 and 3.4 V. For technical reasons, in the case of the measurement of dynamic characteristics of chemical sensors, it is best to obtain activation by a unit step concentration change.

Measurements of characteristics were performed on a measuring stand, the schematic diagram of which is shown in figure 2. The first phase is based on the removal of air from the system and thermal stabilization of all measuring devices. Required flows were set by mass flow controllers and the power supply was switched on (without applying voltage to the sensor). The flow of hydrogen removed air from the measuring cell and capillary. After 15 min, the power supply of the measuring bridge with a sensor was switched on and after the next 15 min recording of the sensor signal was started.

The sensor signal value determined the base line during flow of pure hydrogen through the cell. After 30 min, the four-way valve was reset causing the flow of the hydrogen mixture with air (of required concentration) through the cell. A step change of gas mixture concentration was realized in this way. The measured unbalance voltage of the bridge was the response to excitation of the sensor by a step change of the air concentration in the gas mixture. Resetting of the valve and directing pure hydrogen to the cell was performed after 30 min. The measurement was completed after the next 30 min.

The above procedure was repeated for different sensors at hydrogen flows of 100 and $150\,cm^3\,min^{-1}$ and for respective sensor supply voltages. To increase the accuracy of the method, the dynamic characteristic of the sensor was measured twice, once after the step increase (from zero air concentration in hydrogen to a set 6%) and then a decrease (from 6 to 0% by volume of air in hydrogen). The means of both measurements were taken as parameters.

In figures 3 and 4, sets of characteristics show the course of signal changes of sensors: CityTech and ZEG, as the result of step excitation at different gas flows and for various sensor power supply voltages.

For comparison, in figure 5, characteristics have been presented of a dynamic thermal conductivity sensor, measured in identical conditions as for ZEG and CityTech pellistors. Only the power supply voltage changed (adjusted to electric parameters of the TCD sensor).

The obtained characteristics correspond approximately to the response to step excitation of the inert sensor of the first type. Dynamic parameters in the first approximation have been determined for such a sensor.

### Results

### *Dynamic parameters of pellistor sensors*

The response of a type I inert system to step excitation is described by the following function:

$$C(t) = \begin{cases} C_p & \text{dla } t \le 0 \\ (C_k - C_p)\cdot\left(1 - \exp\left(-\frac{t}{\tau}\right)\right) + C_p & \text{dla } t > 0 \end{cases} \tag{5}$$

where $C_p$ is the initial volume concentration of component in mixture, $C_k$ is the final volume concentration of component in mixture and $\tau$ is the time constant of the sensor system (time after which the sensor output signal attains 63% of the final value).

In the case of a real sensor placed in the measurement cell connected by capillaries with the step concentration

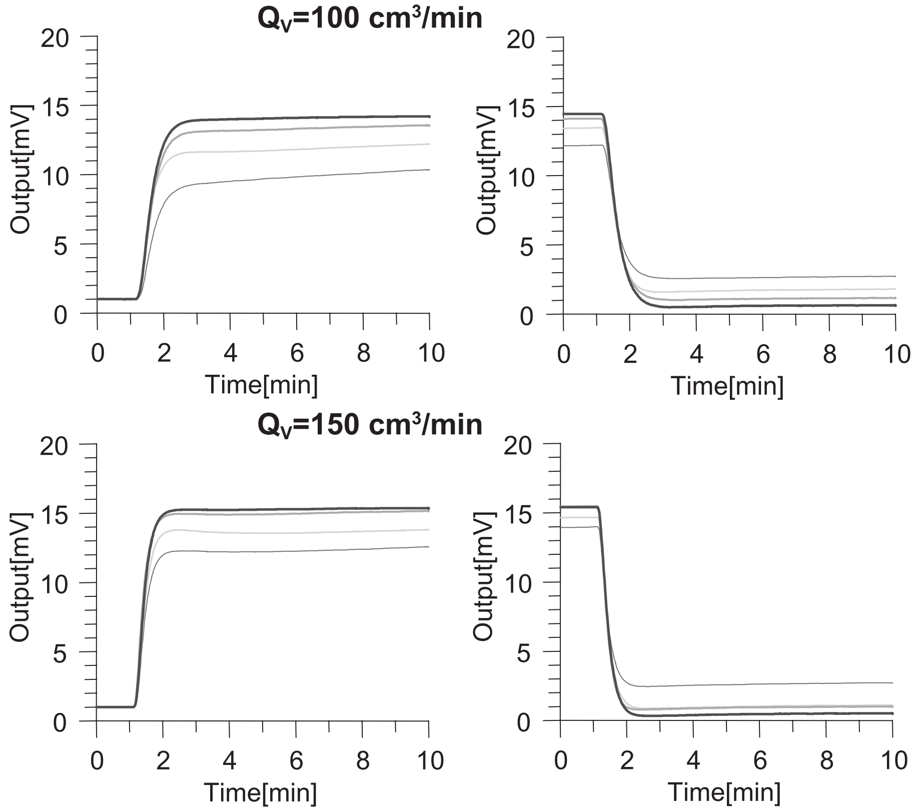

*Figure 3. CityTech sensor response changes after step excitation by air concentration changes (6% by volume) in a gas mixture for the following pellistor power supply voltages, (V): ——, 2.8; ——, 3.0; ——, 3.2; ——, 3.4.*

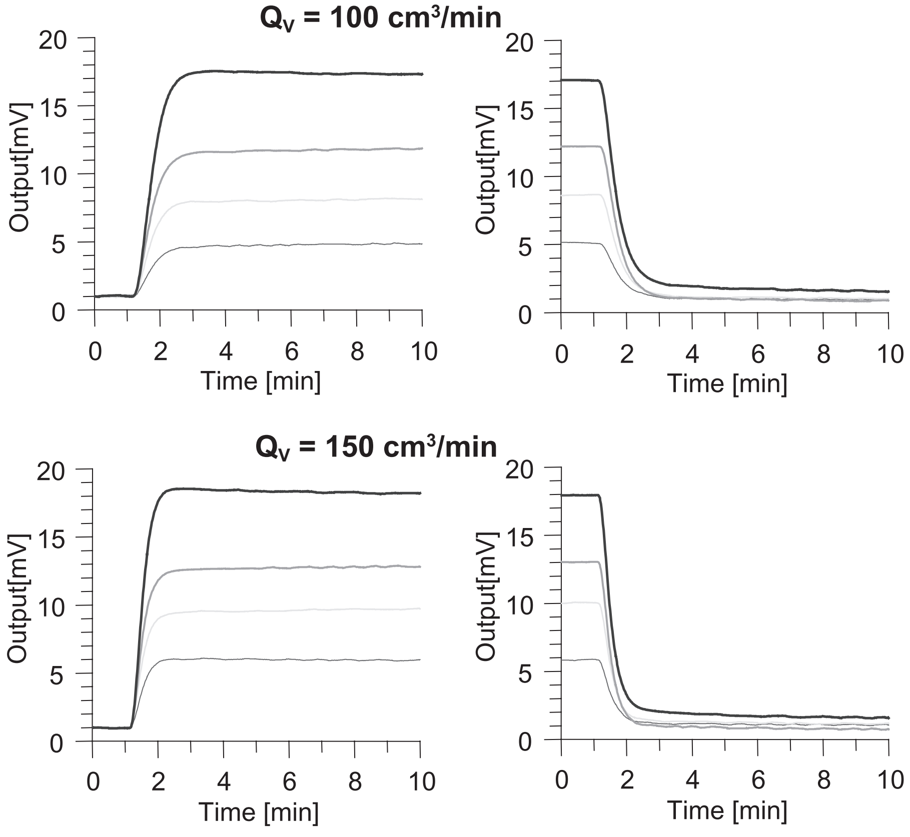

*Figure 4. ZEG sensor response changes after step excitation by air concentration changes (6% by volume) in a gas mixture for the following pellistor power supply voltages, (V): ——, 1.5; ——,1.7; ——,1.9; ——, 2.1.*

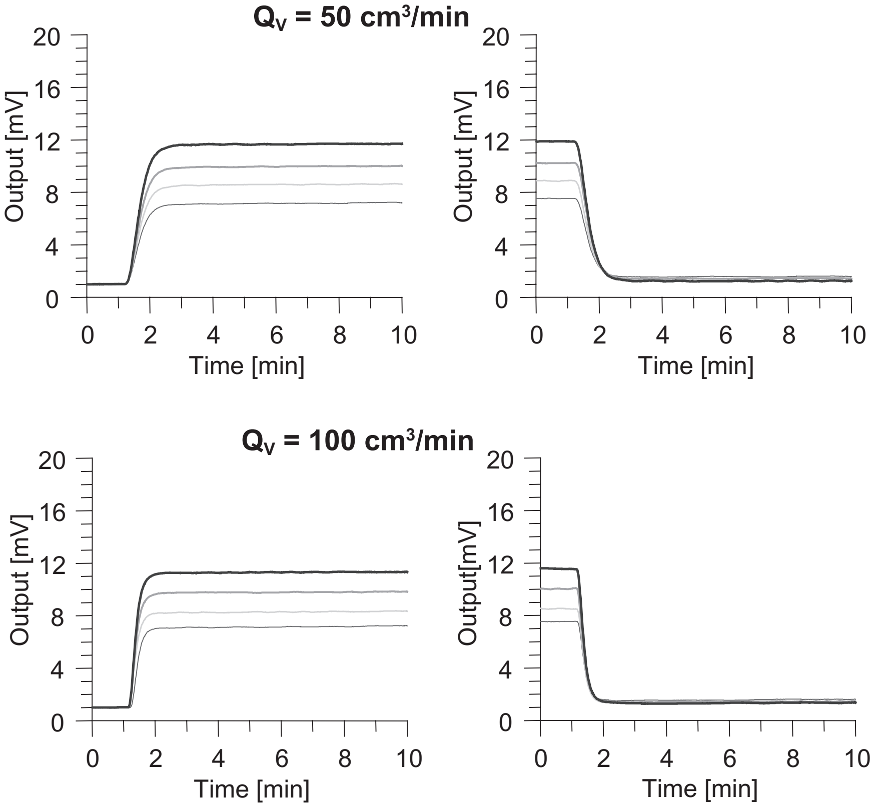

*Figure 5. Thermal conductivity sensor response changes after step excitation by air concentration changes (6% by volume) in a gas mixture for the following sensor power supply voltages: ——, 1.7; ——,1.9; ——, 2.1; ——, 2.3.*

increase excitation system, the dead volume should be taken into account, which is responsible for dead time, i.e., the transport delay ($t_m$) of the gas sample. By modifying the function describing the change of sensor concentration $C(t)$ for a step increase of air concentration in hydrogen from lower to higher values, so as to take into account the dead time the following formula was obtained:

$$C(t) = \begin{cases} C_p \quad \text{dla } t \leq t_m \\ (C_k - C_p)\cdot\left(1-\exp\left(-\frac{t-t_m}{\tau}\right)\right) + C_p \quad \text{dla } t > t_m. \end{cases} \tag{6}$$

Analogously, one may describe function $C'(t)$, where concentration changes from higher to lower values:

$$C'(t) = \begin{cases} C'_p \quad \text{dla } t \leq t'_m \\ (C'_p - C'_k)\cdot\exp\left(-\frac{t-t'_m}{\tau'}\right) + C'_k \quad \text{dla } t > t'_m. \end{cases} \tag{7}$$

Determination of the parameters of sensor response functions $C(t)$ and $C'(t)$ presented in figures 3–5 was performed using the non-linear regression method [18]. Additionally, based on the determined characteristics, sensor sensitivities, $S$, have been calculated for different power supply voltages and at different gas stream flows. Time constants, $\tau$, and dead time, $t_m$, collected in table 2 are means from 'upwards' and 'downwards' sensor excitation.

*Table 2. Experimentally determined dynamic parameter values for: (a) CityTech sensor; (b) ZEG sensor; and (c) TCD sensor.*

| $Q_V$ | $U_z$ [V] | $t_m$ [s] | $\tau$ [s] | S [mV/%] |
|---|---|---|---|---|
| (a) | | | | |
| 100 ($cm^3\,min^{-1}$) | 2.8 | 20 | 27 | 1.30 |
| | 3.0 | 19 | 21 | 1.50 |
| | 3.2 | 19 | 22 | 1.60 |
| | 3.4 | 19 | 22 | 1.70 |
| 150 ($cm^3\,min^{-1}$) | 2.8 | 15 | 15 | 1.55 |
| | 3.0 | 15 | 14 | 1.65 |
| | 3.2 | 15 | 14 | 1.74 |
| | 3.4 | 16 | 15 | 1.80 |
| (b) | | | | |
| 100 ($cm^3\,min^{-1}$) | 1.5 | 21 | 31 | 0.50 |
| | 1.7 | 22 | 28 | 0.95 |
| | 1.9 | 22 | 28 | 1.40 |
| | 2.1 | 23 | 27 | 2.00 |
| 150 ($cm^3\,min^{-1}$) | 1.5 | 17 | 20 | 0.60 |
| | 1.7 | 17 | 20 | 1.10 |
| | 1.9 | 17 | 19 | 1.50 |
| | 2.1 | 18 | 18 | 2.15 |
| (c) | | | | |
| 50 ($cm^3\,min^{-1}$) | 1.7 | 25 | 20 | 1.04 |
| | 1.9 | 25 | 20 | 1.28 |
| | 2.1 | 25 | 20 | 1.51 |
| | 2.3 | 25 | 21 | 1.79 |
| 100 ($cm^3\,min^{-1}$) | 1.7 | 17 | 11 | 1.00 |
| | 1.9 | 16 | 11 | 1.23 |
| | 1.9 | 15 | 11 | 1.48 |
| | 2.3 | 15 | 11 | 1.73 |

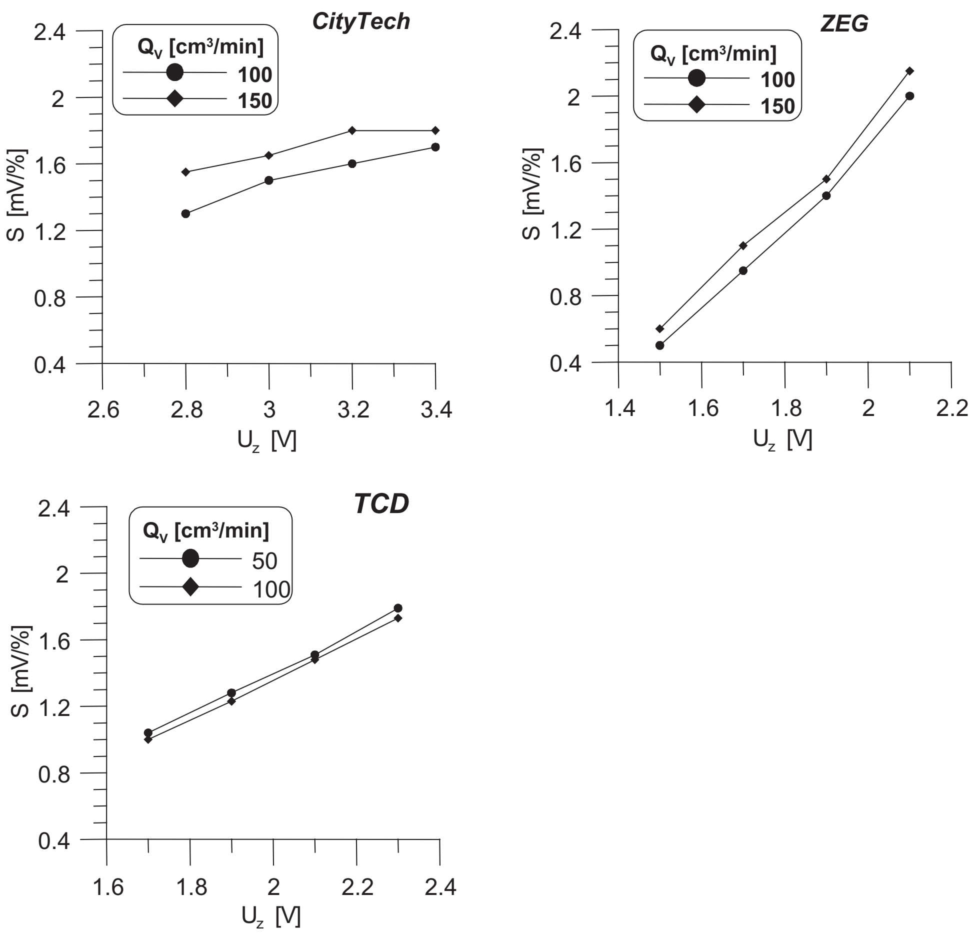

*Figure 6. Relation of CityTech, ZEG and TCD sensor sensitivity versus power supply voltage (for different gas stream flows).*

The analysed mixture stream flow, as expected, has an effect on transport delay. A higher value causes a decrease of transport delay. Similarly, the time constant decreases with an increase of flow. More complex relations are observed in the case of sensor sensitivity.

*Sensor sensitivity in the UEL range*

In figure 6, pellistor sensitivity changes are shown depending on power supply voltages and for different standard mixture flows. Based on the above curves, it can be stated that the sensitivity of the ZEG sensor does not depend substantially on gas stream flow. In addition, this type of sensor is characterized by highest sensitivity. An additional advantage is a low supply voltage.

Owing to a low value of slope of characteristics, it can be concluded that the CityTech sensor signal depends on small changes in the power supply voltage. On the other hand, a small effect is noticed of gas stream flow on sensor sensitivities. Simultaneously, sensitivity is smaller for the ZEG pellistor and attains a maximum at a power supply of about 3.2 V. Large curve slopes show that although the highest sensitivity is obtained for the highest power supply voltage, it is not the optimum ZEG sensor-operating voltage.

The conductivity sensor is characterized by lowest sensitivity, while at the same time it is distinguished by the greatest linearity of sensitivity changes in the function of power supply voltage.

### Conclusions

Conclusions about the application of catalytic combustion sensors for the determination of air in the UEL range are as follows:

- Application of pellistor sensors for investigations of gas mixtures in the hydrogen UEL range is possible but requires determination of optimal operating parameters, primarily of the sensor power supply voltage.
- Sensitivity of pellistors in the UEL range is lower than in the LEL, but is two times higher in the UEL range (in the case of ZEG) than in the case of the thermal conductivity sensor used for comparison.
- Response time of sensors in the UEL range is also lower than in the LEL range. The conductivity sensor used for comparison was characterized by the lowest value, while the CityTech had the lowest value among pellistors and its response time increased by several per cent in the UEL range.

From the above conclusions, although the traditional hydrogen purity measurement method fulfils its role in the case of two-component mixtures, in systems that are more complex and require high reliability, it may be advantageous to use an additional analyser with pellistor sensors acting on a different basis than the thermal conductivity sensor. A possibility of coupling in one measurement system of two types of analytical methods would ensure greater operation safety and better control of industrial processes in which hydrogen is used, e.g. in the production of electric energy.

### Acknowledgement

Part of the investigations was financed by a grant from the State Committee for Scientific Research entitled ‘A new type of analyser for continuous measurement of the upper explosive limit of gas mixtures’ (June 2001–June 2002).

### References

1. Laudyn, D., Pawlik, H. and Strzelczyk, F., *Elektrownie (Powerplants)*, 4th edn (Warsaw: WNT, 2000), 287.
2. Willard, H. H., Merritt, L. L., Dean, J. A. and Settle, F. A., *Instrumental Methods of Analysis*, 6th edn (Belmont: Wadsworth, 1981), 465.
3. Lochmuller, C. H. and Gordon, B. M., A systematic approach to thermal conductivity detector design. *J. Chrom. Sci.*, **16** (1983), 523.
4. Dabill, D. W., Gentry, S. J. and Walsh, P. T., A fast response catalytic sensor for flammable gases. *Sens. Actuators*, **11** (1987), 135.
5. Weast, R. C. (ed.), *Handbook of Chemistry and Physics*, 54th edn (Cleveland: CRC Press, 1974), D-221.
6. Logothethis, E. M., Hurley, M. D., Kaiser, W. J. and Yao, Y. C., Selective methane sensors, in Proceedings of the 2nd International Meeting on Chemical Sensors, Bordeaux, France, 1986, 175.
7. City Technology Ltd, Portsmouth PO6 1SZ, UK [http://www.citytech.com].
8. European Standard EN 50014, *Electrical Apparatus for Potentially Explosive Atmospheres. General Requirements*, 1992.
9. European Standard EN 50018, *Electrical Apparatus for Potentially Explosive Atmospheres. Flameproof Enclosure ‘d’*, 1994.
10. CSA Std C22. 2 No 30-M1986.
11. Zakład Elektroniki Gorniczej ‘ZEG’ S.A., ul. Biskupa Burschego 3, 43-100 Tychy, Poland [http://www.zeg.pl].
12. Namiesnik, J. and Krawczyk, M., Proba zastosowania sensorow katalitycznych do ciagłego pomiaru gornej granicy wybuchowości wodoru. *Pomiary — Automatyka — Kontrola*, **5** (2001), 15.
13. Namiesnik, J., and Krawczyk, M., Analizator z czujnikiem katalitycznym do ciagłego monitorowania wybuchowosci mieszanin gazowych. *Inzynieria i Aparatura Chemiczna*, **4** (2001), 9.
14. Messer Griesheim GmbH, Fütingsweg, 34 4 7805 Krefeld [http://www.messergroup.com]
15. Omega Engineering, INC., Stamford, Connecticut 06907-0047, PO Box 4047, USA [http://www.omega.com]
16. Hameg GmbH, Industriestraße 6, D-63533 Mainhausen, Germany [http://www.hameg.com]
17. Thomas, H., and Fenoglio, J., A stand-alone measurement plotting system. *Hewlett-Packard Journal*, (January 1986), 20.
18. Marquardt, D. W., An algorithm for least squares estimation of parameters. *J. Soc. Ind. Appl. Math.*, **4** (1963), 431.

